# Automated Gait Assessment for Rehabilitation Training Using Pose Tracking and Dynamic Time Warping

**DOI:** 10.3390/diagnostics16081164

**Published:** 2026-04-14

**Authors:** Naomi Yagi, Kazuki Otsuka, Yuki Yamanaka, Kentaro Mori, Yutaka Hata, Yasumitsu Fujii, Yoshitada Sakai

**Affiliations:** 1 Advanced Medical Engineering Research Institute, University of Hyogo, Himeji 670-0836, Japan; 2 Graduate School of Information Science, University of Hyogo, Kobe 650-0047, Japanhata@gsis.u-hyogo.ac.jp (Y.H.); 3 Faculty of Health Care Science, Himeji Dokkyo University, Himeji 670-8524, Japan; yukiyama@gm.himeji-du.ac.jp; 4 Department of Electrical and Computer Engineering, National Institute of Technology, Maizuru College, Maizuru 625-8511, Japan; k.mori@maizuru-ct.ac.jp; 5 Department of Rehabilitation Medicine, Ishikawa Hospital, Himeji 671-0221, Japan; 6 Division of Rehabilitation Medicine, Kobe University Graduate School of Medicine, Kobe 650-0017, Japan; yossie@med.kobe-u.ac.jp

**Keywords:** markerless analysis, walking evaluation, joint angle, rehabilitation, artificial intelligence, motion capture

## Abstract

**Background:** In rehabilitation medicine, efficient gait analysis is crucial for evaluating postoperative recovery and frailty, especially given the increasing burden on clinicians due to an aging population. **Objectives:** This study aims to conduct preliminary validation of an automated linear walking evaluation system using 2D AI posture tracking. By evaluating the basic accuracy of the system on healthy individuals, we aim to establish a technical foundation for future introduction into clinical rehabilitation settings. **Methods:** In this observational study, we utilized a standard visible light camera for practical use. To evaluate accuracy, we compared 2D AI tracking against a gold-standard three-dimensional (3D) motion capture system during normal walking trials with 10 healthy participants. Specifically, we employed Dynamic Time Warping (DTW) to temporally align the asynchronous data streams from the 2D and 3D systems, ensuring precise comparison of joint angles. **Results:** Following the DTW-based alignment, the similarity with the 3D system was 0.806 ± 0.094 overall (Left: 0.797 ± 0.101, Right: 0.814 ± 0.086). **Conclusions:** In this preliminary validation, the proposed 2D AI posture tracking showed good agreement with the gold standard 3D motion capture for gait in healthy individuals. While the average systematic bias was within clinically acceptable limits, the observed limits of agreement suggest that this system is currently optimal as a foundational tool for gait screening. These results establish a technical foundation for the clinical application of this system.

## 1. Introduction

Currently, the aging population in the world is rapidly progressing, and population aging is expected to progress even more rapidly in the future in developed and developing regions. In recent years, the aging rate, which is the proportion of individuals aged ≥65 years in the total world population, has increased from 5.1% in 1950 to 9.4% in 2020 and is expected to further increase to 18.7% in 2060. Over the next 40 years, the population is expected to age rapidly [1]. In Japan, aging is also a serious problem, and the aging rate, which was 28.6% in 2020, is expected to continue increasing and reach 37.9% in 2060. Additionally, a survey by the Ministry of Health, Labor, and Welfare in Japan reported that the number of patients with fractures, which is a typical injury and disease requiring rehabilitation treatment, has been recently increasing and that a higher proportion of patients with fractures are older adults [2]. Consequently, as the population ages, the number of patients undergoing rehabilitation due to fractures, arthritis, and musculoskeletal disorders is expected to increase; reducing the burden on medical professionals in rehabilitation is very important.

Quantitative human motion analysis methods include measurement using acceleration sensors and motion capture. However, these methods pose problems and disadvantages, including requiring large and expensive equipment for exclusive use, limited locations, and complicated preparations for measurement. It is rarely used in routine clinical practice due to the time-consuming procedure of attaching markers to the patient’s body [3,4]. Furthermore, attaching markers can be invasive and may alter a patient’s natural gait pattern. As a result, it is often impractical to adopt these conventional systems into routine rehabilitation. Therefore, there is a strong demand for a markerless, low-cost, portable system that can easily provide objective gait parameters in hospital wards and corridors. To address this need, several recent studies on human skeleton estimation using videos have been actively conducted. Previous studies included various approaches using stereo cameras [5], multi-view cameras [6,7], and depth images [8], volume prediction method [9]; and the method using a convolution module [10]. Historically, markerless motion capture relied on depth sensors, such as Microsoft Kinect, to estimate joint centers [11]. While these systems offered a low-cost alternative to optical motion capture, they required specialized hardware, had low sampling rates, and limited outdoor applicability. In recent years, the field has undergone a paradigm shift with the emergence of deep learning and convolutional neural networks (CNNs). Algorithms such as OpenPose [12] and DeepLabCut [13] enable pose estimation from standard 2D RGB video without the need for depth information or markers. This advancement has popularized gait analysis, enabling retrospective analysis of existing footage and remote patient monitoring using consumer cameras.

The most typical training evaluation in rehabilitation is gait evaluation. It is mainly performed visually by doctors and physical therapists; therefore, it requires plenty of time and effort, and improving efficiency is required. In contrast, the automatic evaluation using video analysis not only reduces the burden on specialists but also quantifies the evaluation. Additionally, it helps realize remote consultations by capturing, sending, and receiving videos using the currently widespread smartphones and tablets. We developed a gait analysis support system to perform quantitative evaluation by calculating the gait features using the OpenPose, human tracking, or floor reaction force meter in our previous studies [14,15,16,17]. However, there is no evidence regarding the relationship between OpenPose and motion capture system, which is the gold standard for gait assessment. Although OpenPose has rapidly gained popularity across various fields, its application to clinical gait analysis remains under validation. Previous studies have evaluated the concurrent validity of OpenPose with gold-standard systems [18,19]. However, results have been inconsistent. While sagittal plane hip kinematics often show high correlation, accuracy for knee and ankle joints varies significantly across studies due to factors such as camera viewpoint, clothing, and occlusion. Furthermore, many existing studies focus on simple static postures or gait in controlled environments, lacking detailed analysis of error characteristics essential for clinical interpretation.

Therefore, rigorous technical validation to clarify the limits of agreement for specific gait parameters is urgently needed. A major methodological challenge in validating such systems is the temporal discrepancy in gait cycles. Observed gait patterns often differ significantly from representative data in terms of walking speed and rhythmic consistency. In such cases, simple point-to-point (frame-by-frame) error comparison is invalid due to phase shifts [20,21]. Therefore, to rigorously evaluate the accuracy of a markerless system, simply comparing spatial coordinate data is insufficient; an analysis method that considers discrepancies on the time axis, which means temporal consistency, is essential.

This study aims to validate the clinical application of a markerless gait analysis system using a monocular camera and OpenPose. The main objective is to evaluate its kinematic accuracy and reliability compared to the gold standard 3D motion capture system, particularly verifying the temporal and spatial consistency necessary for clinical interpretation. Ultimately, the goal is to provide a low-cost quantitative evaluation tool usable in everyday rehabilitation settings.

## 2. Materials and Methods

### 2.1. Participants

The study was conducted in accordance with the Declaration of Helsinki, and approved by the Ethics Committee of Advanced Medical Engineering Research Institute, University of Hyogo, Japan (protocol code 22-005 and date of approval 10 March 2023). Informed consent was obtained from all subjects involved in the study. Subjects were excluded based on the following criteria to eliminate confounding factors affecting gait mechanics:

- History of severe orthopedic diseases in the lower limbs or spine.
- Previous joint replacement surgery (e.g., artificial hip or knee joints).
- Presence of gait disorders caused by neurological conditions, or inability to walk independently without walking aids.

The clinical trial was performed on a total of 10 healthy participants (males 5; females 5) with a mean ± standard deviation (SD) age of 28.7 ± 14.0 (range 20 to 58) years and a height of 164.3 ± 8.0 (range 150.0 to 174.5) cm. Participants were asked to walk 10 m straight three times.

### 2.2. Camera System and Deployment Scheme

**Figure 1:**
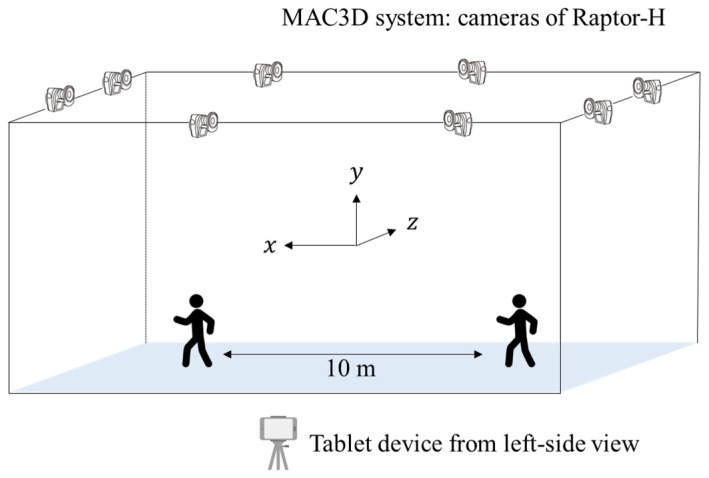
Concurrent measurements for the motion capture of the MAC3D system and tablet devices from a lateral viewpoint.

The concurrent measurements for walking evaluation employ the use of two systems, including (1) the motion capture of MAC3D system (Motion Analysis Corp., Santa Rosa, CA, USA) and (2) the tablet device system of iPad Pro, 1st generation (Apple Inc., Cupertino, CA, USA) (Figure 1).

The motion capture of the MAC3D system comprises eight cameras of Raptor-H with a pixel count of 640 pixels (width), 480 pixels (height), temporal resolution of 240 frames per second (fps indicates the frame rate). Conversely, the iPad Pro has a pixel count of 1920 pixels (width), 1080 pixels (height), and temporal resolution of 60 fps. Details of the digital recording of both systems were indicated as codecs of Windows Media Video (compression coding format developed by Microsoft, Redmond, WA, USA) and the exposure time of 4 to 8 s.

As shown in Figure 1, the iPad Pro was mounted on a tripod at a height of approximately 0.9 m, which corresponds to the approximate center of gravity of an adult human [22,23]. This minimizes perspective distortion of the lower limbs. The camera was positioned a vertical distance of approximately 3.5 m from the center of the 10 m walking path to capture images of the subject from a lateral perspective [24,25]. The rationale for this placement is as follows.

- Clinical practicality: A single-camera lateral capture setup is adaptable to narrow hospital corridors, where multi-view systems are difficult to install. Capturing two steps requires at least 2.5 m. By utilizing corridor intersections and entrances, it can be used even in hospital corridors. Environmental factors related to brightness may affect the accuracy of the estimation.
- Angle standardization: The optical axis was kept perpendicular to the walking direction. This standardization is essential to ensure the accuracy of joint estimation using OpenPose (version 1.5.1), as deviations in walking angle can lead to parallax errors in kinematic calculations.
- Field of View (FOV): The capture distance was determined to record at least two complete gait cycles within a frame while maintaining sufficient pixel resolution for joint center localization.

To ensure robust pose estimation, experiments were conducted in a controlled laboratory environment with blackout curtains to block out fluctuating natural light. The room was lit exclusively by fixed artificial overhead lighting to maintain uniform illumination. Regarding subject appearance, subjects were instructed to wear form-fitting clothing to minimize clothing noise and prevent loose fabric from obscuring anatomical landmarks essential for accurate joint center localization [19]. While no specific color was required, wearing form-fitting clothing enabled the CNN-based algorithm to clearly identify the body contours against a neutral background [12,13].

### 2.3. Measuring Systems

To measure the 3D coordinates of each part of the body, reflective markers (13 mm diameter) were attached to 31 points defined as the Helen Hayes marker set [26,27], which is a regular dataset of markers to represent skeletal model-based analysis, such as joint angles and joint moments. The points comprise 26 front side viewpoints and 5 back side viewpoints. We enabled motion tracking using the motion analysis software of Cortex-64 1.1.4 (Motion Analysis Corp., Santa Rosa, CA, USA).

**Figure 2:**
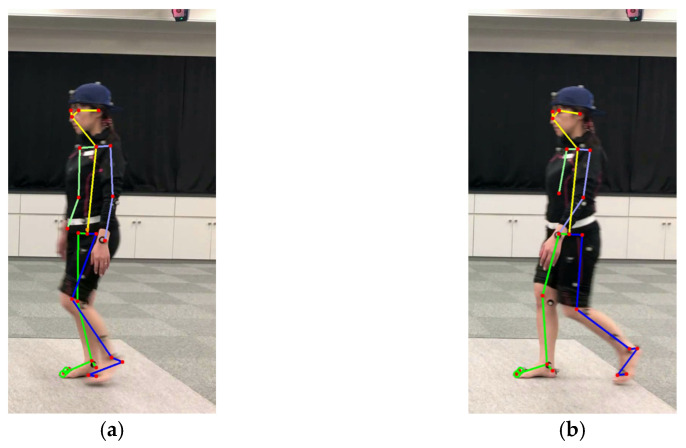
Output examples of OpenPose analysis. (**a**) Successful detection of all anatomical landmarks. (**b**) Incomplete detection with missing points.

To measure the 2D coordinates of each part of the body, OpenPose detects 25 points, encompassing 23 front side body viewpoints and 2 back side body viewpoints. Figure 2 shows an example of analysis output from OpenPose. Figure 2a is an example where all anatomical landmarks were successfully detected, while Figure 2b is an example of incomplete detection where some points are missing. In our experiments, we performed pose tracking using Python (version 2.7.16, Python Software Foundation, Beaverton, OR, USA, www.python.org). Machine specifications are presented in Table 1.

### 2.4. Definitions and Calculation Methods of Joint Angles

**Figure 3:**
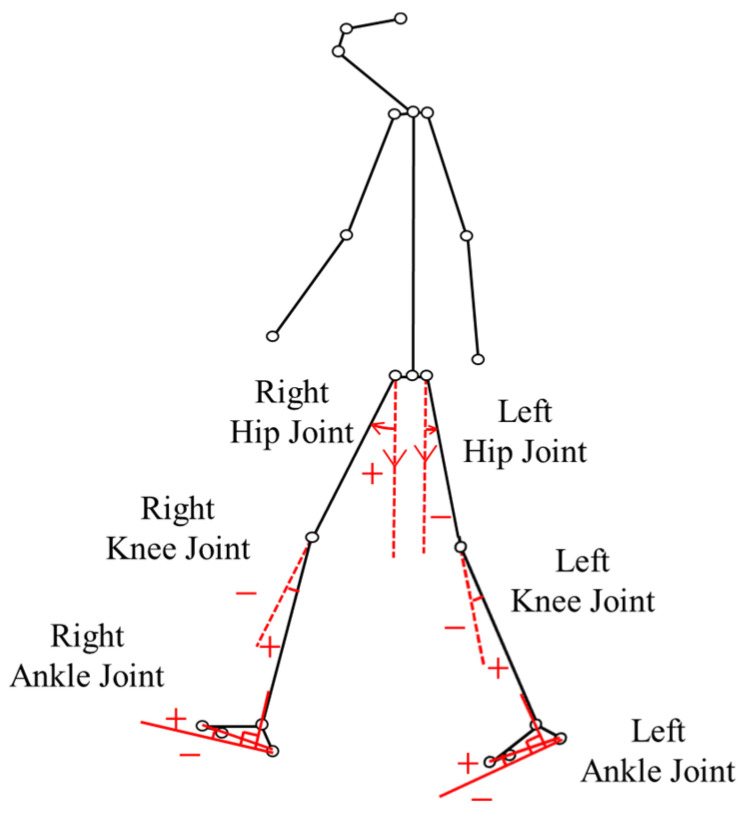
Joint angles of hip, knee, and ankle from left-side lateral view.

To quantitatively evaluate gait mechanics, angular changes in the hip, knee, and ankle joints from left-side lateral view were calculated based on the left lateral view of the subjects (Figure 3). The definitions for each joint angle and the directionality of positive/negative values were established as follows.

- Hip Angle: A vertical line passing downward through the center of the hip joint was defined as the reference axis. The position where the thigh axis aligns with this reference line was defined as 0°. Displacement of the thigh anteriorly (flexion) was calculated as a positive (+) value.
- Knee Angle: The state of full extension, where the thigh axis and the lower leg axis are aligned in a straight line, was defined as the reference 0°. From this position, posterior movement of the lower leg resulting in knee bending (flexion) was calculated as a positive (+) value.
- Ankle Angle: The neutral position, where the lower leg axis and the foot form a right angle (90°) based on the lines connecting the knee, heel, and toe, was defined as the reference 0°. From this reference position, displacement where the toes are lifted toward the knee (dorsiflexion) was calculated as a positive (+) value.

The specific angular parameters analyzed in this study are the continuous time-series kinematic waveforms (flexion/extension) of the hip, knee, and ankle joints for both the left and right sides from left-side lateral view. Rather than extracting discrete points such as the maximum angle, the entire gait cycle dataset was used for statistical comparison.

The OpenPose system calibration involved spatial scaling based on each subject’s standing height. Two-dimensional pixel coordinates were converted to physical dimensions by calculating the ratio of known height to height in pixels.

### 2.5. Similarity Evaluation Based on Signal Processing

We employ DTW [28,29] to evaluate the time-series similarity between joint angle data obtained by motion capture and joint angle data estimated by OpenPose. Let *X* denote the time-series data measured by motion capture, and *Y* denote the time-series data estimated by OpenPose, with data lengths of *N* and *M*, respectively. Here, $x_{i}$ and $y_{i}$ represent a specific joint angle at each frame. Due to sampling rates of each measurement equipment, the data lengths do not match (*N* ≠ *M*).

(1) $$X=\left\{x_{1},\;x_{2},\cdots ,x_{N}\;\right\}$$

(2) $$Y=\left\{y_{1},\;y_{2},\cdots ,y_{M}\;\right\}$$

In DTW, distance calculations are performed to identify optimal correspondence (warping path) between two sequences. First, let $d(x_{i}$, $y_{i})$ denote the local distance between data points $x_{i}$ and $y_{i}$. In this study, we employ the Euclidean distance (absolute value) to represent the difference in angular data.

(3) $$d(x_{i},y_{i})=\left|x_{i}-y_{i}\right|$$

Next, we calculate the cumulative distance D(i, j) from the starting point of the time series to each point (i, j). It is computed recursively based on dynamic programming using the following recurrence formula:

(4) $$D\left(i,\;j\right)\;=\;d\left(x_{i},y_{i}\right)+min(D\left(i-1,\;j\right),\;D\left(i,\;j-1\right),\;D\left(i-1,\;j-1\right))$$

The finally derived cumulative distance D(N, M) is defined as the DTW distance ${DTW}_{dist}$ between the time series *X* and *Y*.

(5) $${DTW}_{dist}=D(N,\;M)$$

Since the calculated ${DTW}_{dist}$ is a cumulative value, it tends to increase depending on length (number of frames) of compared time-series data. Therefore, to eliminate the influence of data length and ensure consistent evaluation across data of varying lengths, we perform normalization based on the length of the warping path (number of steps). The similarity score is calculated using the following exponential function based on a Gaussian kernel (σ=10).

(6) $$Similarity\;Score=exp(-{DTW}_{dist}/\sigma )$$

The Gaussian kernel parameter σ = 10 was selected to optimize the system’s time resolution (60 fps) and the sensitivity of the similarity measure to the assumed noise characteristics of the pose estimation algorithm. According to Müller [30], the kernel width determines the tolerance for phase shift in DTW-based similarity calculations. Since the mean jitter in OpenPose-based gait analysis typically falls within a specific spatial and temporal range [18], this parameter setting effectively acts as a threshold. This setting maintains high sensitivity to macro-level kinematic deviations, which at the current sampling rate corresponds to approximately 15% of the gait cycle, while accommodating intrinsic measurement noise.

### 2.6. Data Analysis

The primary kinematic variables for comparison in this study were the joint angles of the hip, knee, and ankle joints observed from the left side throughout the gait cycle. Specifically, three joints were analyzed: hip (flexion/extension), knee (flexion/extension), and ankle (dorsiflexion/plantarflexion). All joint angles were calculated based on coordinate data from OpenPose and the motion capture system.

**Figure 4:**
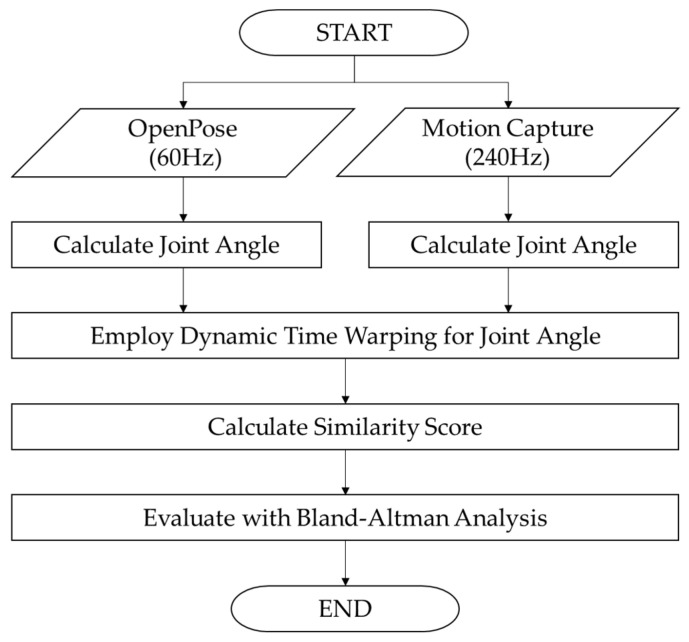
Process flowchart of the proposed gait evaluation system.

Process flowchart of the proposed gait evaluation system is shown in Figure 4. Prior to calculating the kinematic parameters, the raw coordinate data extracted from both systems was preprocessed to ensure data quality. Data synchronization between the two systems was performed based on the coordinates of the left ankle joint point. The moment of ground contact, which shows a characteristic change in motion in the trajectory of the left ankle joint point, was used as a common time reference point. Missing values were identified based on the confidence score provided by OpenPose. The points with a confidence score below 0.45 were deemed unreliable and treated as missing data. This threshold was determined by considering the trade-off between maximizing data retention and minimizing the inclusion of falsely detected joint points. First, missing values due to occlusion or tracking errors were filled using linear interpolation. Only gaps of ≤12 consecutive frames (0.2 s) were filled using linear interpolation; longer gaps resulted in trial exclusion. Outliers were also detected and replaced using linear interpolation. Next, low-pass Butterworth filters at 6 Hz were applied. This frequency was selected based on [22], as it effectively removes high-frequency noise while preserving the dominant frequency content of human gait (typically <5 Hz).

The calculated angle time-series data were aligned using DTW to correct for the time discrepancy between the two systems, and the degree of agreement was calculated. Furthermore, Bland–Altman analysis [31] was used to verify the systematic error (bias) and limits of agreement between the two measurement methods.

Signal processing and data analysis procedures are executed using the MATLAB programming environment (Version R2024a; The MathWorks, Inc., Natick, MA, USA). All statistical analyses are performed using the IBM SPSS Statistics software (version 29.0, IBM Corp., Armonk, NY, USA). A *p* value < 0.05 was considered statistically significant.

Of the 30 trials (10 participants × 3 trials), 2 trials were excluded due to measurement errors in the motion capture system. As a result, the final analysis of the hip and knee joints was based on 28 trials (*n* = 28). Further exclusions were made for the ankle joint due to OpenPose tracking errors, resulting in a final sample size of 27 trials for the left side and 26 trials for the right side.

## 3. Results

### 3.1. Quantitative Similarity Evaluation Using DTW

The data were preprocessed using a 6 Hz low-pass Butterworth filter to minimize high-frequency noise before the DTW and Bland–Altman analyses were performed. Table 2 presents the results of the time-series similarity evaluation and correlation analysis between the 2D joint angles estimated by OpenPose and the 3D joint angles measured by the motion capture system. The overall similarity score across all trials and all joints (hip, knee, and ankle) was 0.806 ± 0.094, indicating a high degree of concordance between the proposed method and the gold standard.

Regarding specific joint performance, the hip joint demonstrated the highest similarity, with an average score of 0.833 ± 0.080 (Left: 0.821 ± 0.094, Right: 0.846 ± 0.100). This was followed by the knee joint at 0.794 ± 0.106, and the ankle joint at 0.779 ± 0.090. Furthermore, regarding laterality, the mean score for the left side was 0.797 ± 0.101, while the right side was 0.814 ± 0.086. These results indicate no significant difference in estimation accuracy between the near side (left) and the far side (right) relative to the camera, suggesting that the proposed single-camera lateral setup can stably evaluate gait movements on both sides.

A one-sample *t*-test was performed for each joint and each side to determine whether the similarity score deviated significantly from zero. Bonferroni correction was applied to address the issue of multiple comparisons across three joints and two sides (*n* = 6), and a significant difference of *p* < 0.0083 (=0.05/6) was observed.

### 3.2. Visual Inspection of Time-Series Waveforms

**Figure 5:**
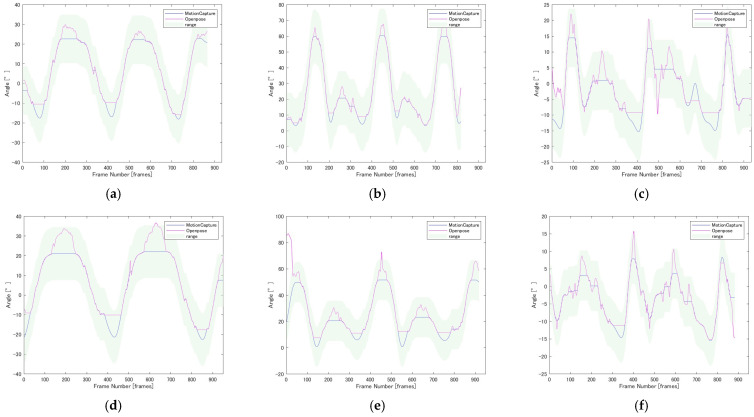
Time-series joint angles aligned by DTW. (**a**) Participant A: Hip Joint Angle, Similarity Score = 0.854; (**b**) Participant A: Knee Joint Angle, Similarity Score = 0.862; (**c**) Participant A: Ankle Joint Angle, Similarity Score = 0.792; (**d**) Participant B: Hip Joint Angle, Similarity Score = 0.714; (**e**) Participant B: Knee Joint Angle, Similarity Score = 0.638; (**f**) Participant B: Ankle Joint Angle, Similarity Score = 0.904.

Figure 5 illustrates representative examples of joint angle waveforms aligned using DTW. The pink lines represent the measured values from the motion capture system, while the green lines represent the estimated values from OpenPose. The light green shaded area indicates the tolerance range, which was defined as ±30% of the maximum physiological range of motion of the joint. Following the temporal normalization process by DTW, the phases of the waveforms from both systems were well-aligned, confirming that the flexion and extension patterns during the gait cycle were accurately captured. In individual cases, such as Participant A (Figure 5a,b), high similarity scores were recorded for hip (0.854) and knee (0.862) joints. Conversely, although some variance was observed, such as in Participant B (Figure 5d–f) where the knee joint score was 0.638, the overall trend confirmed that the waveforms estimated by OpenPose closely followed the behavior of the gold standard system.

### 3.3. Bland–Altman Analysis

Bland–Altman analysis was performed to evaluate the agreement between the motion capture system and OpenPose system for each joint angle (Table 3). It was presented as mean bias and standard deviation. Positive values indicated that OpenPose system overestimated the angle compared to the motion capture system. Systematic bias for all joints was less than 1.6°, demonstrating good agreement overall. Specifically, the mean biases for the hip, knee, and ankle were less than 1.6°, 0.8°, and 1.6° for both the left and right sides, respectively. The standard deviations of the difference between the right knee joint and ankle joint were large, at 4.421 and 4.189, respectively.

A one-sample *t*-test was performed for each joint and side to determine whether the mean bias deviated significantly from zero. To address the issue of multiple comparisons across three joints and two sides (*n* = 6), Bonferroni correction was applied, adjusting the significance level to *p* < 0.0083 (=0.05/6). Due to the large number of data points, statistically significant differences were still observed after Bonferroni correction. While statistically significant, the magnitude of these differences did not exceed the clinical threshold suggested by previous studies.

The 95% Limits of Agreement (LoA) of the right knee joint ranged from −8.120° to 9.210°, reflecting the high variability indicated by a standard deviation of 4.421°. Similarly, the LoA of the right ankle joint also ranged widely from −7.006° to 9.414°.

## 4. Discussion

The accuracy observed in this study is consistent with or even exceeds previous literature findings. McGinley et al. [32] demonstrated that a measurement error of less than 2° is considered good, and an error of 5° or less is acceptable for clinical judgments. The systematic bias of our system falls within good range, and the random error is comparable to the inter-rater variability commonly observed with manual marker placement. Furthermore, recent studies examining the effectiveness of OpenPose for gait analysis, such as Stenum et al. [18] and Mündermann et al. [33], have reported mean absolute errors at lower limb joints ranging from approximately 4.0° to 7.0°. Our results are in good agreement with these benchmarks, confirming that our proposed system achieves the benchmark-level accuracy for single-camera markerless estimation. Systematic bias remained within the good range (less than 2°), but the 95% LoA for the knee and ankle joints reached approximately ±9°. This exceeds the 5° threshold generally recommended for accurate clinical judgment. Such variability is likely due to joint occlusion or jitter in 2D posture estimation, suggesting that this system is better suited to broad clinical screening and identification of broad gait abnormalities than to monitoring subtle postoperative improvements.

Compared to conventional 3D optical motion capture systems, the proposed approach offers significant advantages in terms of feasibility and time-efficiency [3]. Conventional systems require expensive equipment, dedicated laboratory space, and time-consuming marker placement, which can be burdensome for patients. In contrast, our system requires only a commercially available tablet and enables instant data acquisition in any environment, including hospital corridors. Although there is a trade-off of slightly reduced accuracy compared to gold-standard methods, the accessibility and non-invasiveness of our system make it a practical tool for routine screening and long-term monitoring of rehabilitation progress.

In this study, DTW was employed to evaluate the time-series similarity between joint angle data acquired by a motion capture system and those estimated by OpenPose. Generally, camera-based pose estimation is prone to temporal discrepancies compared to ground truth data, caused by processing latency or differences in frame rates. Consequently, simple Euclidean distance, which compares data frame-by-frame, may yield large error values even when waveform shapes are similar, rendering accurate evaluation difficult [20]. In contrast, DTW is a method that determines the optimal alignment by non-linearly warping the time axis of time-series data. This approach enabled a shape-based similarity evaluation that tolerates temporal shifts. This processing step was crucial to separate spatial accuracy from temporal lag.

The right knee joint showed higher error compared to the hip and ankle joints. This decrease in accuracy is likely due to frequent self-occlusion, where the opposing leg temporarily blocks the camera’s line of sight to the knee during mid-stance and mid-swing. Furthermore, the effects of soft tissue artifacts and clothing deformation are more pronounced at the knee. Even with the use of tight-fitting sportswear to minimize this interference, unlike the rigid ankle and stable trunk (hip), the knee is covered by fabric that moves during flexion and extension, which introduces noise into keypoint detection. Finally, systematic offsets due to different definitions of joint centers are also a contributing factor. This is because optical systems rely on tactile bony landmarks (e.g., the lateral epicondyle of the femur), while OpenPose estimates a probabilistic visual center of the joint.

### Study Limitations

The sample size of this study was relatively small (*n* = 10). However, it should be noted that this sample size was determined based on a priori power analysis for a two-tailed Pearson’s correlation test. Assuming a strong correlation (effect size r = 0.80) between the joint angles estimated by the proposed method and the motion capture system, we recruited a sample size of participants necessary to achieve a statistical power of 0.80 (1 − β) at a significance level of 0.05 (α). Assumed variance was not applicable for this correlation-based power analysis. However, we recognize that this sample of healthy young adults (28.7 ± 14.0 years) is not representative of older adults or patients with pathological gait, which is common in rehabilitation. Pathological gait often involves reduced walking speed and compensatory movements, which can impair the tracking performance of visual-based algorithms. Therefore, this study should be interpreted as a preliminary technical validation for establishing a baseline in a controlled environment. While these foundational results are promising, they do not constitute conclusive evidence of clinical utility. Further studies involving diverse clinical populations, such as patients with hemiplegia or joint diseases, are essential to validate the reliability of the system in routine clinical practice.

Furthermore, this study primarily focused on verifying the concurrent validity (accuracy) of this system against a gold standard. However, test–retest reliability was not explicitly evaluated at this stage. Although the use of an automatic pose estimation algorithm (OpenPose) eliminated inter-rater variability associated with manual marker placement [34], this study did not explicitly quantify test–retest reliability (intra- and inter-session reproducibility). For clinical implementation, it is essential to verify whether the system can provide consistent results even when measurements are taken on different days or when different examiners install the cameras. Therefore, future studies should evaluate the system’s reliability using measures such as intraclass correlation coefficients in repeated trials.

## 5. Conclusions

In this study, we demonstrated that a markerless gait analysis system using monocular camera footage can achieve favorable estimation accuracy by applying DTW. The key finding is that lower limb joint angles estimated by OpenPose, following temporal normalization via DTW, exhibited substantial similarity to the optical motion capture system of the gold standard. A robust correlation was confirmed for the knee joint, suggesting its potential as a primary indicator in gait function evaluation.

In conclusion, this study demonstrated that combining the proposed markerless system with DTW yields good estimation accuracy in a controlled environment. The results show a high degree of similarity to the gold standard, suggesting its potential as a screening tool. However, users should be aware that the 95% agreement limit can reach ±9° in certain joints. This may limit its application in scenarios requiring highly accurate individual monitoring. Furthermore, while this study established the simultaneous validity of the system, further investigation is needed regarding test–retest reliability. Demonstrating consistent results through repeated measurements will be an essential next step toward future clinical applications.

## Figures and Tables

**Table 1:** Machine specifications for analysis.

Contents	Details
Central Processing Unit	Intel Core i7-8750H
Operating System	64-bit Windows 10
Random Access Memory	32 GB
Graphics Processing Unit	NVIDIA GeForce RTX 2070

**Table 2:** DTW-based similarity scores and Pearson correlation for joint angles (Hip and Knee: *n* = 28; Left Ankle: *n* = 27; Right Ankle: *n* = 26).

Joint Angle	Side	Similarity Scores [-]	Pearson Correlation [-]
Hip	Left	0.821 ± 0.094 *	0.978 *
	Right	0.846 ± 0.062 *	0.980 *
	Both	0.833 ± 0.080 *	0.979 *
Knee	Left	0.793 ± 0.113 *	0.965 *
	Right	0.794 ± 0.100 *	0.950 *
	Both	0.794 ± 0.106 *	0.958 *
Ankle	Left	0.777 ± 0.095 *	0.904 *
	Right	0.802 ± 0.084 *	0.898 *
	Both	0.789 ± 0.090 *	0.900 *
All	Left	0.797 ± 0.101 *	0.974 *
	Right	0.814 ± 0.086 *	0.969 *
	Both	0.806 ± 0.094 *	0.972 *

* *p* < 0.001.

**Table 3:** Mean bias of difference and 95% LoA in joint angles on Bland–Altman analysis (Hip and Knee: *n* = 28; Left Ankle: *n* = 27; Right Ankle: *n* = 26).

Joint Angle	Side	Mean Bias of Difference [°]	95% LoA [°]
Hip	Left	1.523 ± 3.147 *	−4.645 to 7.691
	Right	0.971 ± 2.994 *	−4.897 to 6.839
Knee	Left	0.773 ± 2.627 *	−4.376 to 5.922
	Right	0.545 ± 4.421 *	−8.120 to 9.210
Ankle	Left	1.528 ± 3.827 *	−5.973 to 9.029
	Right	1.204 ± 4.189 *	−7.006 to 9.414

* *p* < 0.001.

## Data Availability

The original contributions presented in this study are included in the article. Further inquiries can be directed to the corresponding author.
